# Lines Left Uncrossed: The Cost of Attrition in Metastatic Triple Negative Breast Cancer

**DOI:** 10.7759/cureus.107389

**Published:** 2026-04-20

**Authors:** Nandini Devi R, Arun Krishnan M.P, Praveen K Shenoy, Abhilash Menon

**Affiliations:** 1 Medical Oncology, Malabar Cancer Centre, Post Graduate Institute of Oncology Sciences &amp; Research (PGIOSR), Thalassery, IND

**Keywords:** attrition, breast neoplasms, drug therapy/chemotherapy, line of therapy, metastases, survival, tnbc

## Abstract

Background

Treatment attrition poses an important challenge in the management of breast cancer patients worldwide. There is a paucity of data specifically addressing the attrition rates of metastatic triple-negative breast cancer (mTNBC), which has the worst survival among all breast cancer subtypes.

Materials and methods

In this retrospective study, we assessed attrition rates among patients with metastatic TNBC and their causative factors. Additionally, we analyzed the median progression-free survival (PFS) across each line of therapy (LOT) and the median overall survival (OS). Attrition was defined as discontinuation of anti-cancer therapy due to any cause. The case records of all patients with metastatic TNBC treated between January 01, 2017, and December 31, 2021, were screened, and relevant data were extracted in a pre-specified format.

Results

Seventy patients with metastatic TNBC were included for analysis. The median age was 50 years (range: 29-82). About 18 patients (25.7%) experienced attrition before initiating any anticancer therapy for metastatic TNBC. Following each LOT, attrition occurred as follows: 28.5% after the first line, 14.2% after the second line, 5.7% after the third line, and 4.2% after the fourth line. Only two patients received fifth-line therapy; both had attrition. The leading causes for attrition were decline of performance status, followed by disease progression. Median PFS declined with each LOT, and median OS was 14.8 months.

Conclusions

In our study, all patients with metastatic TNBC experienced treatment attrition, with the majority receiving two or fewer LOT. Declining performance status and disease progression were the leading causes for attrition. The median PFS reduced with successive treatment lines, underscoring the need for earlier, more effective treatments.

## Introduction

Breast cancer is the most common malignancy among women worldwide. It remains the leading cause of cancer-related mortality in this population [1]. Triple-negative breast cancer (TNBC) is an aggressive subtype characterized by the absence of estrogen receptor (ER), progesterone receptor (PR), and human epidermal growth factor receptor 2 (HER2) protein, which accounts for 10-20% of all breast cancer cases. TNBC exhibits the worst survival outcomes among all breast cancer subtypes. TNBC predominantly affects younger age females, presents with advanced stages, exhibits aggressive biology, and subsequently leads to poor outcomes. TNBC also imposed a substantial economic and humanistic burden. A systematic review reported that the mean annual direct medical cost per patient ranges from $20,000 to $100,000 for stage I-III disease and from $100,000 to $300,000 for stage IV disease. Indirect costs, including loss of productivity, range from $207 to $1,573 per month. Advanced disease and recurrence further contribute to reduced workforce participation and increased societal burden [2].

The burden of TNBC seems to be higher in Indian women, with reported prevalence reaching up to 31%. The mean age ranged between 45 and 55 years, and a significant proportion of these females presented with advanced stages [3]. Poor socio-economic status, lower educational qualification, advanced stage at presentation, aggressive disease biology, lack of access to proper treatment, and restricted participation in clinical trials are the main reasons for poor survival outcomes of TNBC in our country [4-6].

Treatment attrition was yet another challenge encountered in the management of breast cancer. Attrition was initially described in the context of longitudinal studies. While traditionally defined as loss of participants in longitudinal studies, in real-world oncology practice, attrition represents a continuous phenomenon across multiple lines of therapy. High attrition rates can introduce significant bias in treatment outcomes and affect the interpretation of subsequent lines of therapy. This is particularly relevant in advanced breast cancer [7]. The causes of attrition vary across different regions. In India, attrition rates were high among breast cancer patients. Older age, poor socio-economic status, rural residence, and disease progression were the main reasons for treatment attrition in our country [8]. There is a paucity of data specifically addressing the attrition rates among patients with metastatic TNBC, which is the subgroup with the worst clinical outcomes. Understanding real-world attrition rates can help identify the gaps within our cancer care pathway and guide clinicians/policymakers in implementing corrective measures. In this study, we aim to explore the pattern of attrition among patients with metastatic TNBC treated in our center.

## Materials and methods

This was a retrospective cohort study conducted in a tertiary cancer center in South India. The primary objective was to determine the attrition rates and their causative factors among metastatic TNBC patients. The secondary objectives were to determine the median progression-free survival (PFS) associated with each line of therapy (LOT) and to estimate the median overall survival (OS). In addition, we assessed the predictive ability of known prognostic factors for OS. All patients with metastatic TNBC treated in our center between January 01, 2017, and December 31, 2021, were considered eligible for this study. The case records of these patients were screened, and relevant data were extracted in a pre-specified format. Those with incomplete medical records were excluded. The following operational definitions were used:

Attrition: It was defined as the discontinuation of anti-cancer therapy due to any cause. These included referral to the local palliative care center for best supportive care, transfer of treatment to another hospital, lost to follow-up, and death [9].

Progression-free survival (PFS): It was defined as the time interval between the date of initiation of a specific line of systemic anticancer therapy and the date of documented clinical or radiological progression, or death from any cause, whichever occurred first. Sequential PFS intervals were designated as PFS1, PFS2, PFS3, and so forth, corresponding to each respective LOT.

Overall survival (OS): It was defined as the time period from the date of diagnosis of stage IV TNBC to the date of death or last follow-up.

Triple-negative breast cancer (TNBC): It was diagnosed by immunohistochemistry, showing the absence of expression of ER, PR, and HER2 protein.

HER2 low breast cancer: It was diagnosed by immunohistochemistry, showing HER2 scores of 1+ and 2+ with fluorescence in situ hybridization (FISH)-negative.

Patients with metastatic TNBC were treated according to standard treatment guidelines. These patients received sequential systemic therapy on disease progression if they were physically fit. If unfit due to any medical causes, the same was documented in the file, and these patients were referred to the local palliative center for the best supportive care. Palliative radiation therapy was administered whenever clinically indicated. Only systemic therapy was counted as LOT. All patients who were not on regular treatment/follow-up at our center on the date of the data cut-off were considered as attrition. If the reason for attrition is not documented in the patient's file, he/she were telephonically contacted, and the reason for their attrition and survival status were enquired and recorded. This study was performed in line with the principles of Helsinki and approved by our Institutional Review Board (1616/1RB-SRC/13/08-02-2025/3).

Statistical Analysis

Statistical analysis was done using Statistical Product and Service Solutions (SPSS, version 27; IBM SPSS Statistics for Windows, Armonk, NY). Baseline patient demographics and treatment details were summarized using descriptive statistics. Attrition rates were described as a proportion in percentage. The reverse Kaplan-Meier method was used for calculating the median follow-up duration. Kaplan-Meier analysis was used to estimate PFS and OS. The log-rank test was used to compare OS curves across subgroups defined by established prognostic factors such as age at diagnosis, low HER2 status, de novo/recurrent disease, presence of brain metastases, presence of visceral disease such as liver/lung metastases, receipt of palliative radiation, and PFS (less than six months vs more than six months in each LOT). To explore associations between clinical variables and OS, univariate and multivariate Cox proportional hazards models were performed. A p-value <0.05 was considered significant.

## Results

Out of 3,049 breast cancer patients registered at our center between January 01, 2017, and December 31, 2021, around 293 (9.6%) had TNBC. Among them, 70 (23.8%) patients underwent treatment for stage IV disease and were considered for analysis. The median age was 50 years (range: 29-82). No male patients were identified. Low HER2 expression was seen in 20 patients (28.5%), and 21 patients (30%) had de novo metastatic disease. Brain metastases were present in 21 patients (30%), and four patients (5.7%) had leptomeningeal involvement. The baseline characteristics are summarized in Table 1.

**Table 1:** Baseline clinical and demographic characteristics of patient with stage IV TNBC treated between January 1, 2017, and December 31, 2021 * Taxane includes paclitaxel (weekly and three-weekly schedules) and docetaxel HER2: human epidermal growth factor receptor 2; TNBC: triple-negative breast cancer

Variable	Details	N=70 (%)
Median age	In years (range)	50 (29-82)
Type of metastatic disease	De novo	21 (30%)
	Recurrent disease	49 (70%)
HER2 low subtype	-	20 (28.5%)
Site of metastases	Brain	21 (30%)
	Leptomeningeal	4 (5.7%)
	Bone	33 (47.1%)
	Lung	29 (41.4%)
	Pleural deposit/effusion	6 (8.5%)
	Liver	14 (20%)
	Non-regional node	23 (32.8%)
Palliative radiation therapy (n=40)	Whole brain radiation	19 (27.1%)
	Spinal Cord compression	1 (1.4%)
	Painful bone metastases	14 (20%)
	Bleeding breast tumor	10 (14.2%)
Palliative chemotherapy (n=39)	Taxane*	22 (31.4%)
	Capecitabine	21 (30%)
	Gemcitabine-Carboplatin	10 (14.2%)
	Eribulin	4 (5.7%)
	Adriamycin	4 (5.7%)

Attrition Rates and Their Causative Factors

**Figure 1:**
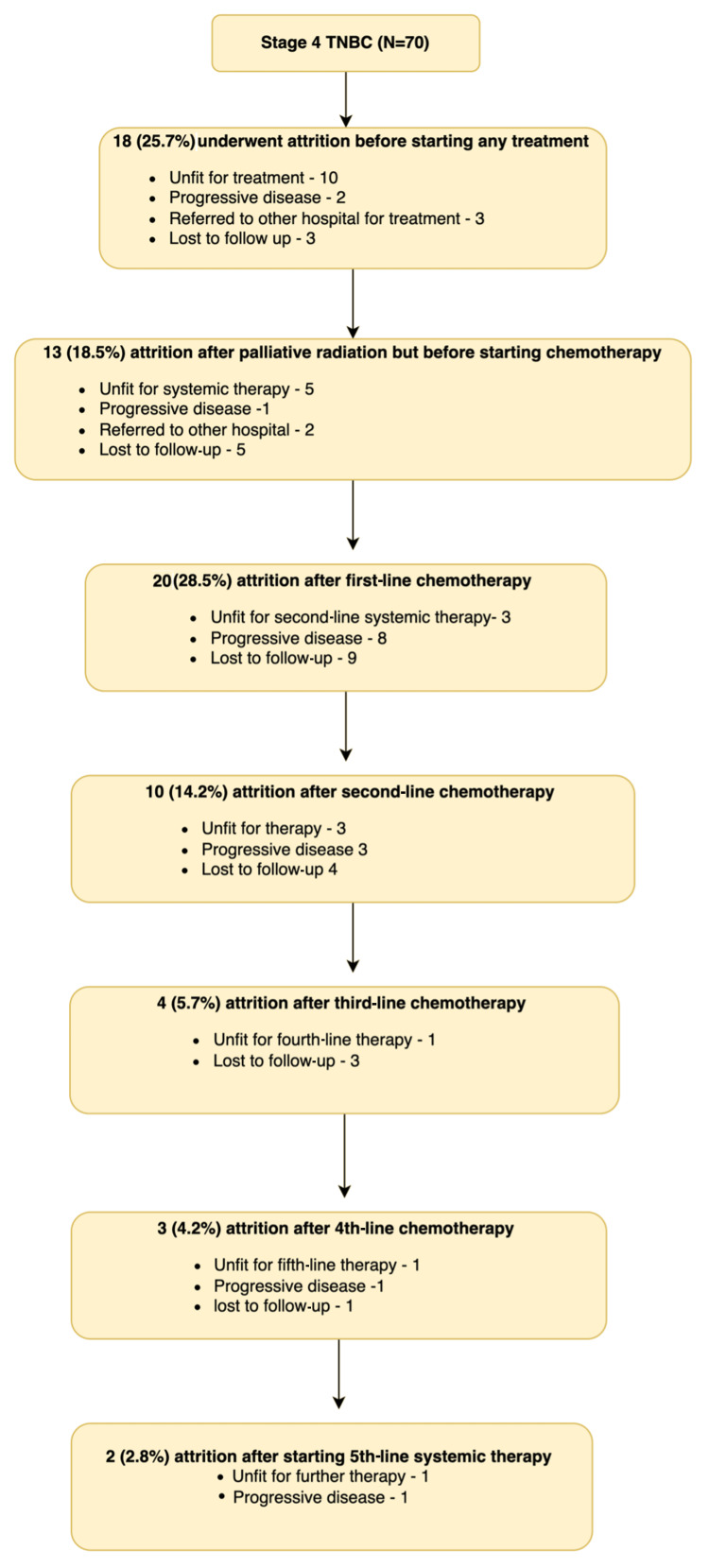
Attrition rates and their causative factors among patients with stage IV TNBC treated between January 1, 2017, and December 31, 2021 TNBC: triple-negative breast cancer

Around 18 patients (25.7%) experienced attrition before initiating any anticancer therapy for metastatic TNBC. Following each line of therapy, attrition occurred as follows: 28.5% after the first line, 14.2% after the second line, 5.7% after the third line, and 4.2% after the fourth line. Only two patients received fifth-line therapy, and both did not complete planned therapy and had attrition. Overall, 87.1% received two or fewer lines of therapy. The leading causes for attrition were decline of performance status rendering patients unfit for further anticancer therapy, followed by progressive disease (Figure 1).

Association Between Established Prognostic Factors and OS

None of the evaluated prognostic factors - including age at diagnosis, low HER2 status, presence of brain metastases, presence of visceral metastases in liver/lung, de novo or recurrent disease, receipt of palliative radiation, lines of therapy (2 vs 3 or more ), PFS1 more than six months or PFS2 more than six months had statistically significant association with OS on log-rank testing and Cox regression analysis.

Survival Outcomes

**Figure 2:**
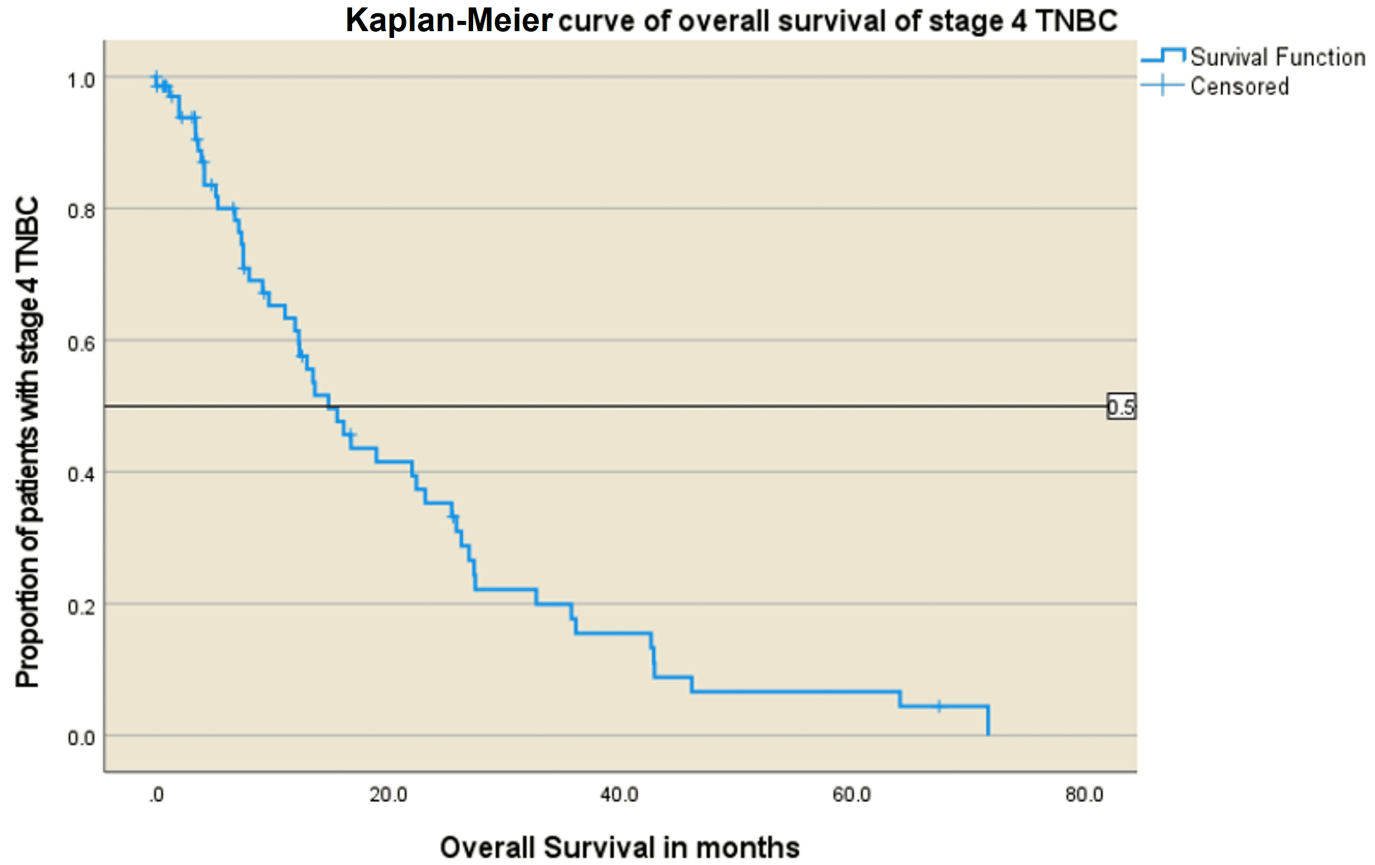
Kaplan-Meier curve of the overall survival of patients with stage IV TNBC TNBC: triple-negative breast cancer

Median follow-up duration was 47 months. Median PFS declined with each line of therapy: PFS1 - 9.9 months, PFS2 - 6.8 months, PFS3 - 2.2 months, and PFS4 - 1.6 months. Median OS was 14.8 months (Figure 2).

## Discussion

This retrospective study explored the attrition rates and their causative factors among patients with metastatic TNBC treated in our centre. The majority of the patients (87%) had attrition within two lines of therapy. Progressive decline in performance status and disease progression were the main reasons for attrition. None of the established prognostic factors was found to be predictive of the OS in this group. Median PFS declined with each line of therapy. Median OS was 14 months after the diagnosis of stage IV disease.

Attrition rates in metastatic breast cancer in Western countries ranged from 9% to 53%, with the majority between 13% and 33%. Most of these studies show high attrition rates in TNBC patients, except the German Praegnant Registry data, which quoted the HER2-positive subset to have the highest attrition rates. Real-world data from the USA report that, despite the rampant use of immune checkpoint inhibitor (ICI) therapy, the attrition rates in metastatic TNBC remained high [7,10-12]. Indian data also align with this trend; stage IV breast cancer patients had 46% attrition rates, though not specifically TNBC [8]. Our study showed even higher attrition rates - 72.8% after first-line therapy. The majority (87%) of our patients experienced attrition within the first two lines of therapy. This sharply contrasts the trend in luminal breast cancers, where patients receive multiple lines of therapy [13]. This underscores the need for providing more effective therapies early in the treatment course for metastatic TNBC to improve survival outcomes and reduce attrition.

In the Italian registry data, the causative factors for attrition are disease progression or death. The authors report that Italian patients have universal insurance coverage for all standard therapies, and, hence, attrition due to inaccessibility is less likely [10]. Real-world evidence from the Flatiron Health Database in the USA also showed that treatment patterns and attrition rate were similar across all socio-demographic groups, but the survival outcomes were uniformly poor [14]. However, the Indian scenario is the exact opposite. Patients from a poor socio-economic background or lower educational status had higher attrition rates. Additionally, access to novel therapies is very limited [8]. Over the five-year time frame, all our metastatic TNBC patients received sequential chemotherapeutic agents only. None of them received ICIs or poly (ADP-ribose) polymerase (PARP) inhibitors approved for TNBC at that time, mainly due to financial constraints. However, since the majority of cytotoxic chemotherapies were covered under government-funded insurance schemes, all patients deemed clinically fit were initiated on chemotherapy [15]. Consequently, the primary causes of attrition in our setting were a decline in performance status and disease progression.

A key strength of our study is that it reflects real-world data from a government tertiary cancer centre situated in a tier-2 city in India. Given this setting, our patient population is broadly representative of the majority of breast cancer patients in the country. We suggest that improving the accessibility and availability of effective and newer targeted therapies approved for TNBC - such as trastuzumab deruxtecan, sacituzumab govitecan, and ICIs - may help reduce attrition rates in our setting. Additionally, increasing awareness about ongoing clinical trials and active patient participation in these trials could improve access to novel therapies, thereby improving treatment outcomes.

The retrospective nature of our study is our major limitation. Through telephone conversations, we could only retrieve the information regarding survival status. We were not able to trace the cause of attrition in 25 patients (35.7%), which constitutes almost a third of this cohort. Socio-economic demographics had been pointed out as a major causative factor for attrition in other Indian studies [8]. Since ours was a retrospective study, a formal assessment of the socio-economic status was not available. The small sample size was also a major drawback, and this might have led to null findings in log-rank and regression analysis.

## Conclusions

Attrition is very high among metastatic TNBC patients in our setting, mainly due to declining performance status and rapid disease progression. None of the known prognostic factors were predictive of the OS in this real-world cohort. The median PFS reduced with successive treatment lines, underscoring the need for earlier, more effective therapeutic strategies.
